# Systemic Inflammation-Associated Proteins and Retinopathy of Prematurity in Infants Born Before the 28th Week of Gestation

**DOI:** 10.1167/iovs.17-21931

**Published:** 2017-12

**Authors:** Mari Holm, Tora S. Morken, Raina N. Fichorova, Deborah K. VanderVeen, Elizabeth N. Allred, Olaf Dammann, Alan Leviton, for the ELGAN Study Neonatology and Ophthalmology Committees

**Affiliations:** 1 Department of Clinical and Molecular Medicine, Faculty of Medicine, Norwegian University of Science and Technology, Trondheim, Norway; 2 Department of Pediatrics, St. Olavs Hospital, Trondheim University Hospital, Trondheim, Norway; 3 Department of Neuromedicine and Movement Science (INB), Faculty of Medicine, Norwegian University of Science and Technology, Trondheim, Norway; 4 Department of Ophthalmology, St. Olavs Hospital, Trondheim University Hospital, Trondheim, Norway; 5 Laboratory of Genital Tract Biology, Department of Obstetrics, Gynecology, and Reproductive Biology, Brigham and Women's Hospital, Harvard Medical School, Boston, Massachusetts, United States; 6 Department of Ophthalmology, Children's Hospital Boston, Boston, Massachusetts, United States; 7 Department of Ophthalmology, Harvard Medical School, Harvard University, Boston, Massachusetts, United States; 8 Department of Neurology, Boston Children's Hospital, Boston, Massachusetts, United States; 9 Department of Neurology, Harvard Medical School, Harvard University, Boston, Massachusetts, United States; 10 Department of Public Health and Community Medicine, Tufts University School of Medicine, Boston, Massachusetts, United States; 11 Perinatal Epidemiology Unit, Department of Gynecology and Obstetrics, Hannover Medical School, Hannover, Germany

**Keywords:** retinopathy of prematurity, biomarkers, preterm birth, inflammation

## Abstract

**Purpose:**

To assess the association between systemic levels of inflammation-associated proteins and severe retinopathy of prematurity (ROP) in extremely preterm infants.

**Methods:**

We collected whole blood on filter paper on postnatal days 1, 7, 14, 21, and 28 from 1205 infants born before the 28th week of gestation, and measured the concentrations of 27 inflammation-associated, angiogenic, and neurotrophic proteins. We calculated odds ratios with 95% confidence intervals for the association between top quartile concentrations of each protein and prethreshold ROP.

**Results:**

During the first three weeks after birth, high concentrations of VEGF-R1, myeloperoxidase (MPO), IL-8, intercellular adhesion molecule (ICAM)-1, matrix metalloproteinase 9, erythropoietin, TNF-α, and basic fibroblast growth factor were associated with an increased risk for prethreshold ROP. On day 28, high levels of serum amyloid A, MPO, IL-6, TNF-α, TNF-R1/-R2, IL-8, and ICAM-1 were associated with an increased risk. Top quartile concentrations of the proinflammatory cytokines TNF-α and IL-6 were associated with increased risks of ROP when levels of neuroprotective proteins and growth factors, including BDNF, insulin-like growth factor 1, IGFBP-1, VEGFR-1 and -2, ANG-1 and PlGF, were not in the top quartile. In contrast, high concentrations of NT-4 and BDNF appeared protective only in infants without elevated inflammatory mediators.

**Conclusions:**

Systemic inflammation during the first postnatal month was associated with an increased risk of prethreshold ROP. Elevated concentrations of growth factors, angiogenic proteins, and neurotrophins appeared to modulate this risk, and were capable of reducing the risk even in the absence of systemic inflammation.

Retinopathy of prematurity (ROP) blinds approximately 20,000 infants annually.^1^ The incidence of ROP in high income countries has remained stable over the last 3 decades,^2^ but due to increased survival of infants born preterm**,** the number of children affected by ROP is rising in low- and middle-income countries.^1^ In addition to the risk of a poor visual outcome, infants with ROP are at increased risk of dysfunctions associated with nonvisual neural disabilities.^3–6^ These co-occurrences suggest a shared etiology with similar risk factors for retinopathy and brain damage in premature newborns.^3,7^

In the extremely low gestational age newborn (ELGAN) study^8^ systemic inflammation has been associated with damage to the lung, bowel, and brain.^9–11^ Increasing evidence points to an association between neonatal inflammation and ROP.^12–17^ The dysregulation of retinal vascular development is an essential factor in ROP pathogenesis.^18^ Angiogenesis and inflammation are closely related, and molecules such as erythropoietin (EPO); VEGF; insulin-like growth factor-1 (IGF-1); and angiopoietins play multiple roles in neural, vascular, and inflammatory processes.^19–23^ The role of these proteins in systemic circulation in ROP etiology remains unclear.^18,24,25^

To our knowledge, no study has evaluated the risk of ROP in light of the relationship between potential sources of damage such as circulating inflammation-related proteins and potential protectors (e.g., IGF-1 and neurotrophins) during the entire first neonatal month in a large cohort. The ELGAN study provides an opportunity to evaluate these relationships.

## Methods

### Participants (Table 1)

The ELGAN study is a prospective observational cohort study of children born before the 28th week of gestation at 1 of 14 participating hospitals in five states in the United States during the years 2002 through 2004.^8^ Mothers were approached for informed consent either upon antenatal admission or shortly after delivery. A total of 1506 infants born to 1249 mothers were enrolled. Infants with major birth defects or/and aneuploidy were excluded. The 1205 children who had at least one eye exam and one or more blood spots collected during the first postnatal month comprise the sample for this report. The institutional review boards of all participating institutions approved the enrollment and consent processes. The research presented here adheres to the tenets of the Declaration of Helsinki.

**Table 1:**
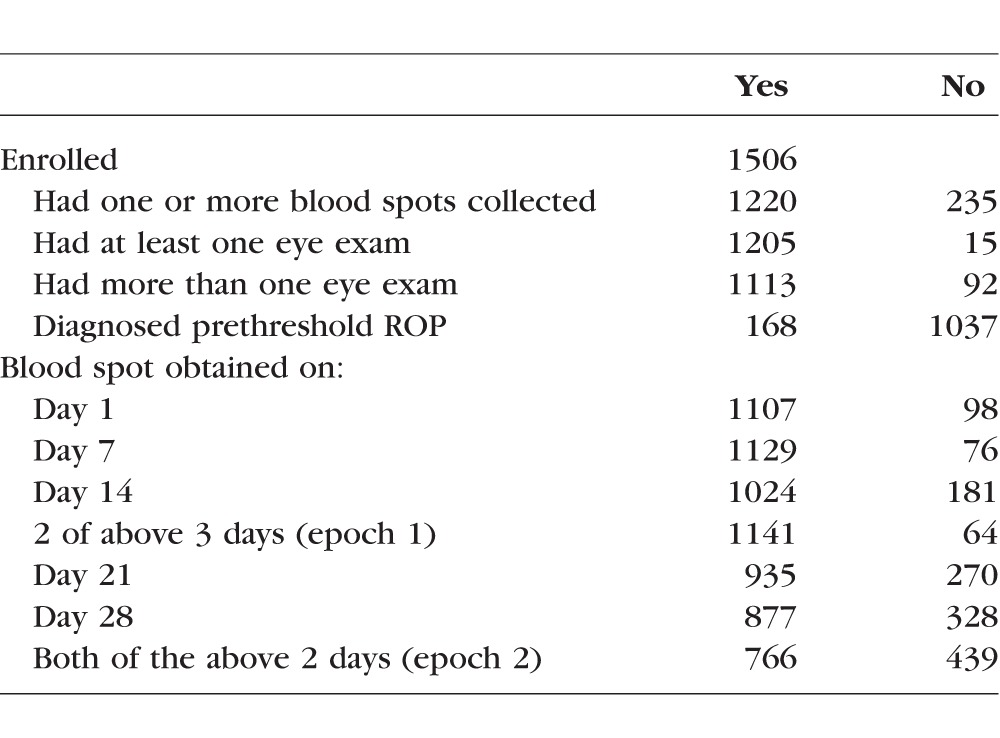
Sample Description

### Newborn Variables (Table 2)

Estimate of gestational age was based on date of embryo retrieval, intrauterine insemination, or fetal ultrasound before the 14th week (62%). When any of these were not available, the estimate was based on fetal ultrasound at week 14 or later (29%); last menstrual period (7%); or gestational age recorded in the log of the neonatal intensive care unit (1%). Birth weight z**-**score was defined as the number of standard deviations above or below the median weight of infants of the same gestational age in a referent samples not delivered for preeclampsia or fetal indications.^26^ Infants with birth weight z-score between −2 and −1 were defined as moderately growth restricted, and infants with a z-score below −2 were defined as severely growth restricted.

**Table 2:**
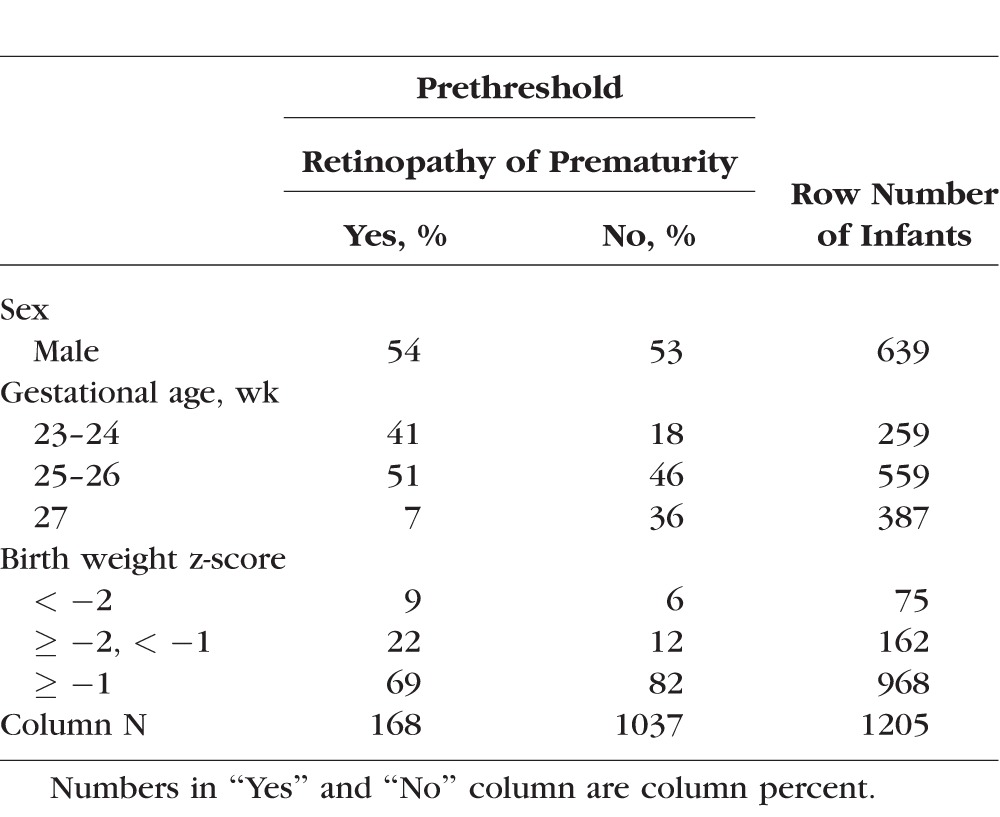
Characteristics of Infants in the Sample

### Eye Exam

Definitions of ROP stages were those accepted by the International Committee for Classification of Retinopathy of Prematurity.^27^ In keeping with guidelines, the first ophthalmologic examination was within the 31st to 33rd postmenstrual week.^28^ Follow-up exams were as clinically indicated until normal vascularization began in zone III, and the most severe ROP stage was recorded. We focused our analyses on prethreshold ROP, defined as any ROP in zone I, ROP stage 2 or 3 with plus disease, or ROP stage 3 without plus disease in zone II.^29^

### Blood Spot Collection and Protein Measurement

Drops of blood were collected on filter paper (Schleicher & Schuell 903) on postnatal days 1 (range: 1–3 days); 7 (range: 5–8 days); 14 (range: 12–15 days); 21 (range: 19–23 days); and 28 (range: 26–29). All blood was obtained from the remainder of specimens obtained for clinical indications. Dried blood spots were stored at −70**°**C in sealed bags with a desiccant until processed. Details of the process of elution of protein from blood spots are provided elsewhere.^30^ The total protein concentration in each eluted sample was determined by bicinchoninic acid assay (Thermo Fisher Scientific, Inc., Rockford, IL, USA) using a multi-label counter (Victor 2; Perkin Elmer, Boston, MA, USA). The concentrations of each protein listed in Table 3 and measured as detailed below were normalized to milligrams of total protein.

**Table 3:**
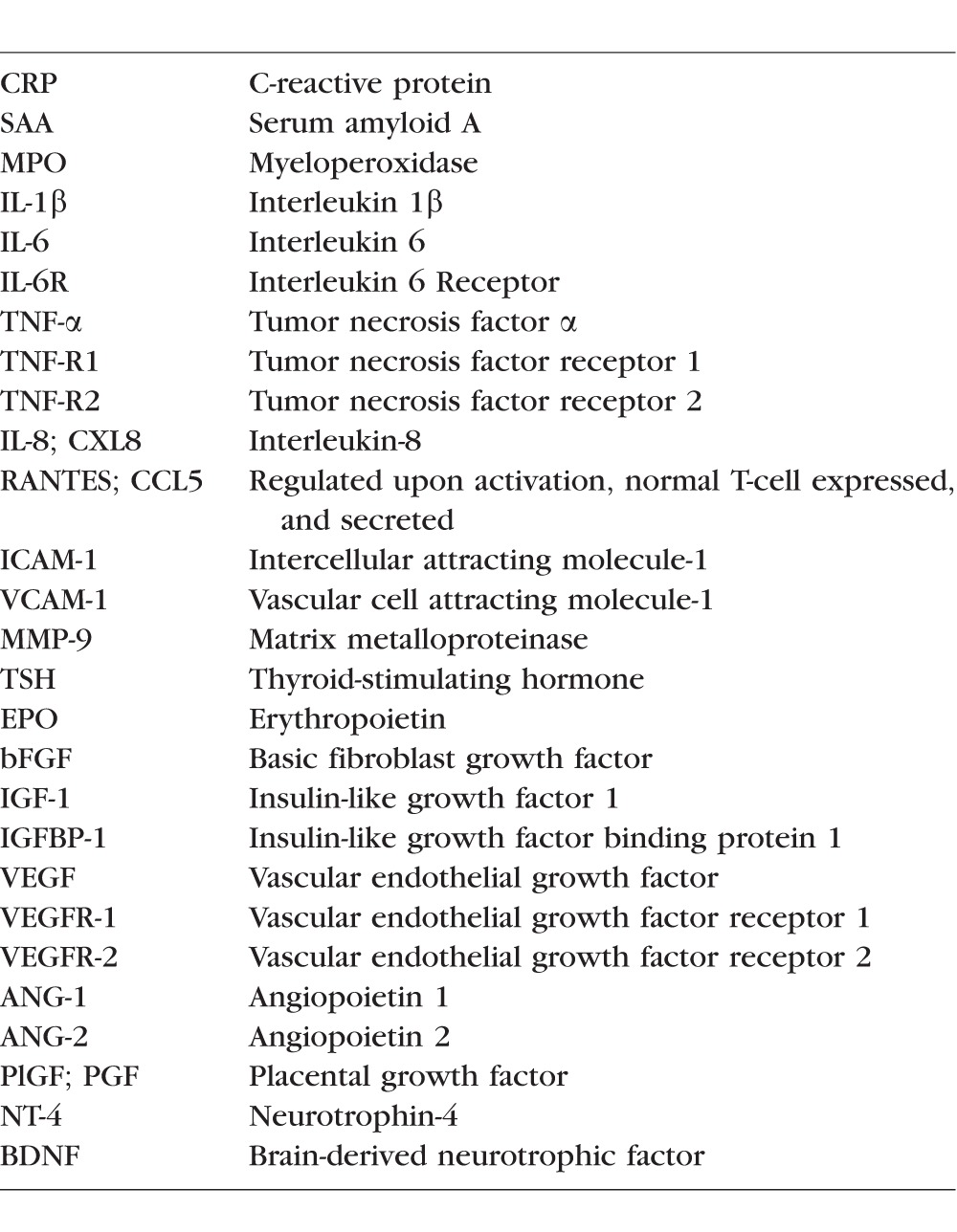
Proteins Measured on Days 1, 7, 14, 21, and 28

### Protein Biomarker Measurements (Table 3)

All proteins listed in Table 3 were measured at the Genital Tract Biology Laboratory at the Brigham and Women's Hospital in Boston Massachusetts. The procedure is described in detail elsewhere.^31^ The laboratory used meso scale discovery multiplex platform and microplate detection platform (Sector Imager 2400; Gaithersburg, MD, USA) to measure C-reactive protein (CRP); serum amyloid A (SAA); myloeperoxidase (MPO) interleukin (IL)-1β; IL-6, IL-6 Receptor (IL-6R); TNF-α, TNF receptor-1 (TNF-R1); TNFR-2; IL-8 (CXCL8); regulated upon activation, normal T-cell expressed, and secreted (RANTES; CCL5); intercellular adhesion molecule-1 (ICAM-1; CD54); vascular cell adhesion molecule-1 VCAM-1; CD106); VEGF; VEGF receptor-1 (VEGFR-1; sFLT-1); VEGFR-2 (KDR); IGF-1 binding protein-1 (IGFBP-1); thyroid stimulating hormone (TSH); matrix metalloproteinase-9 (MMP-9); and EPO. A multiplex immunobead assay manufactured By R&D Systems (Minneapolis, MN, USA) and a commercial reader (MAGPIX Luminex; R&D Systems) were used to measure angiopoietin 1 (ANG-1); angiopoietin 2 (ANG-2); placenta growth factor (PlGF); neurotrophin-4 (NT-4); brain-derived neurotrophic factor (BDNF); and basic fibroblastic growth factor (bFGF). ELISA (R&D Systems) was used to measure IGF-1.

The concentrations of proteins measured in the ELGAN Study varied with gestational age category (23–24, 25–26, 27 weeks), and postnatal day of blood collection (1, 7, 14, 21, and 28).^30,32^ Because we were interested in the contribution of both high and low concentrations, and the concentrations of most proteins did not follow a normal distribution, the distribution of each protein's concentration was divided into quartiles.

The proteins included were analyzed in two sets years apart with an “early epoch” of specimens (days 1, 7, and 14) measured from 2010 to 2011, and a “late epoch” (days 21 and 28) from 2014 to 2015. Consequently, the two different sets were analyzed separately. We divided our sample into 30 groups defined by gestational age category (23–24, 25–26, 27 weeks), postnatal day of blood collection (1, 7, 14, 21, and 28), and measurement set (2009–2010, 2015). The infants were classified as being in the top quartile or not compared to their peers in in the same group.

### Data Analyses

We tested the hypothesis that infants who had a protein concentration in the top quartile on each day were no more likely than their peers to be given a diagnosis of prethreshold ROP. In this sample, both low gestational age and fetal growth restriction are associated with protein concentrations,^30,33^ as well as with prethreshold ROP.^34^ Consequently, we adjusted for gestational age category (23–24, 25–26, 27 weeks) and birthweight z-score < −1. Stratification by gestational age would have led to reduced statistical power. We created logistic regression models to evaluate each biomarker individually. These models allowed us to calculate odds ratios (OR) and 95% confidence interval (CI). Risk ratios cannot be calculated in logistic regression models, and thus cannot adjust for important confounders such as gestational age and birthweight.

Early sustained inflammation was defined as a protein concentration in the top quartile on two or more of the first three protocol days (days 1, 7, and 14), and late sustained inflammation was defined as being in the top quartile on both of the last two protocol days (days 21 and 28). Assessments for sustained/recurrent inflammation were restricted to children who had blood collected on 2 or 3 days in the “early epoch”, and on both days in the “late epoch.”

High concentrations of neurotrophic and/or angiogenic proteins have the potential to prevent brain damage and enhance repair.^35^ We therefore conducted additional analyses examining ROP risk in light of the concurrent concentrations of two proteins, one with pro-inflammatory properties, and one with neurotrophic properties. In these logistic regression analyses children who did not have high (i.e., top quartile) concentrations of either protein were the referent group.

## Results

In this sample of infants selected on the basis of gestational age at birth, the incidence of prethreshold was 27% among children born during weeks 23 or 24, 14% among children born during the 25th or 26th weeks, and only 3% among children born during the 27th week of gestation.

### Early Epoch: Days 1, 7, and 14 (Table 4)

The risk of prethreshold ROP risk was increased when day 1 concentrations of VEGFR-1 were in the top quartile and day 14 concentrations of MPO, IL-8, ICAM-1, MMP-9, and EPO were in the top quartile. In contrast, a reduced ROP risk was associated with top quartile concentrations of the following: day 1, SAA; day 7, VEGF and ANG-1; and day-14, RANTES, TSH, BDNF, and ANG-1. Recurrent/sustained elevated concentrations (i.e., being in the top quartile on ≥2 days) of MPO and IL-8 in the early epoch (i.e., days 1, 7, and 14) were associated with increased ROP risk. A recurrent/sustained elevated concentrations of RANTES were associated with decreased risk.

**Table 4:**
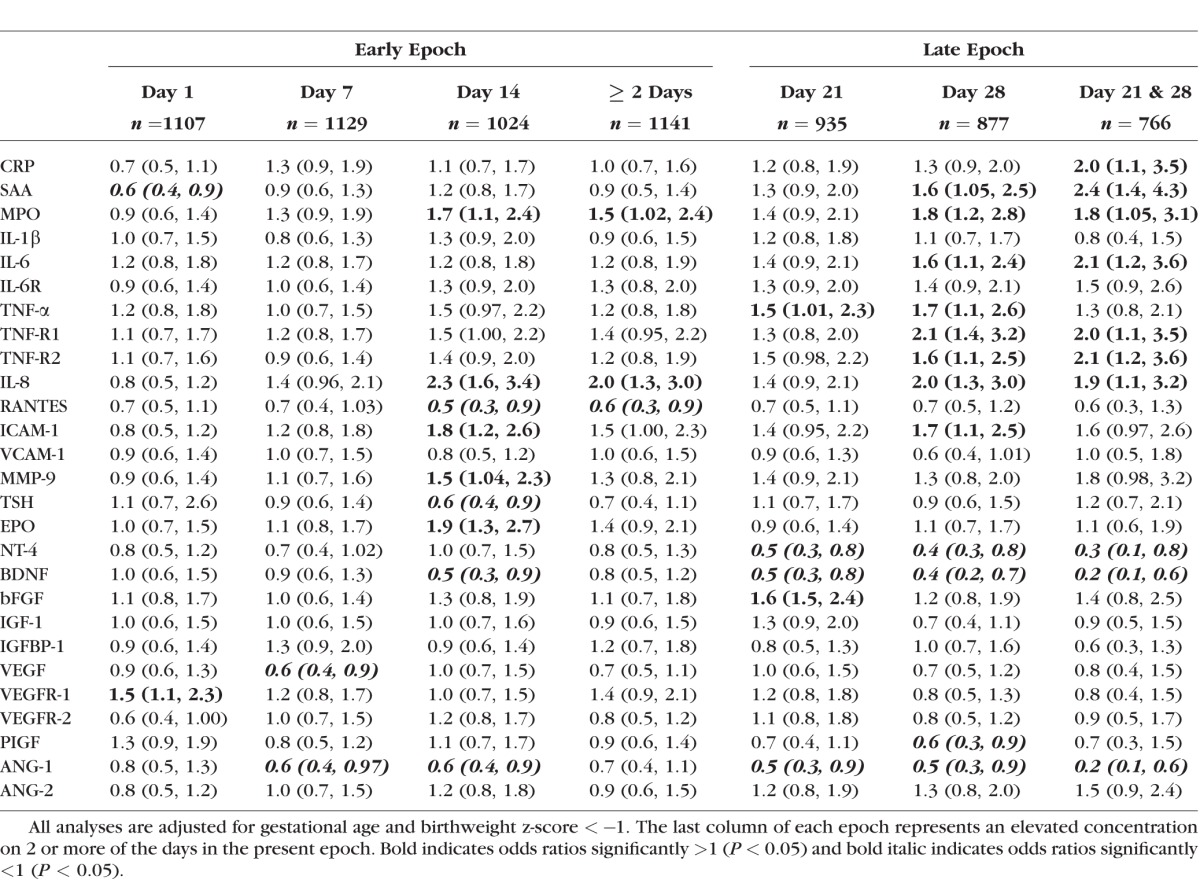
Odds Ratios (95% CI) for Prethreshold ROP (Number of infants, n = 168) Comparing Children With a Top Quartile Concentration of the Protein on the Left on Each Postnatal Day to Children in the Same Gestational Age Category Who Had a Concentration in the Lower Three Quartiles on the Corresponding Day

### Late Epoch: Days 21 and 28 (Table 4)

In the late epoch, an increased risk of prethreshold ROP was associated with a top quartile of the following proteins: day 21 TNF-α and bFGF, and day 28 SAA, MPO, IL-6, TNF-α, TNF-R1 and -R2, IL-8, and ICAM-1. In contrast, the top quartile concentrations of BDNF, NT-4 and ANG-1 on day 21 and 28, and PIGF on day 28 only, were associated with a reduced ROP risk.

Recurrent/sustained elevated late epoch concentrations (i.e., in the top quartile on both days 21 and 28) of CRP, SAA, MPO, IL-6, TNF-R1, TNF-R2, and IL-8 were associated with increased ROP risk. A reduced risk was associated with recurrent/sustained elevations of NT-4, BDNF, and ANG-1.

### ROP Risk Related to the Co-occurrence of Proteins on Day 28 (Table 5)

We wanted to study how the co-occurrence of presumed “protective” and “damaging” biomarkers influenced the risk of prethreshold ROP. In Table 5, the Ang-2 × IL-6 +/+ group has an OR of 2.0; since the lower bound of 95% CI does not include 1.0, the OR is significantly elevated compared to the referent group (−/−) at the *P* < 0.05 level. Conversely, the NT-4 × IL-6 (+/−) OR is 0.4; since the upper bound of the 95% CI does not include 1.0, the OR is significantly reduced compared to the referent group (−/−) at the *P* < 0.05 level.

**Table 5:**
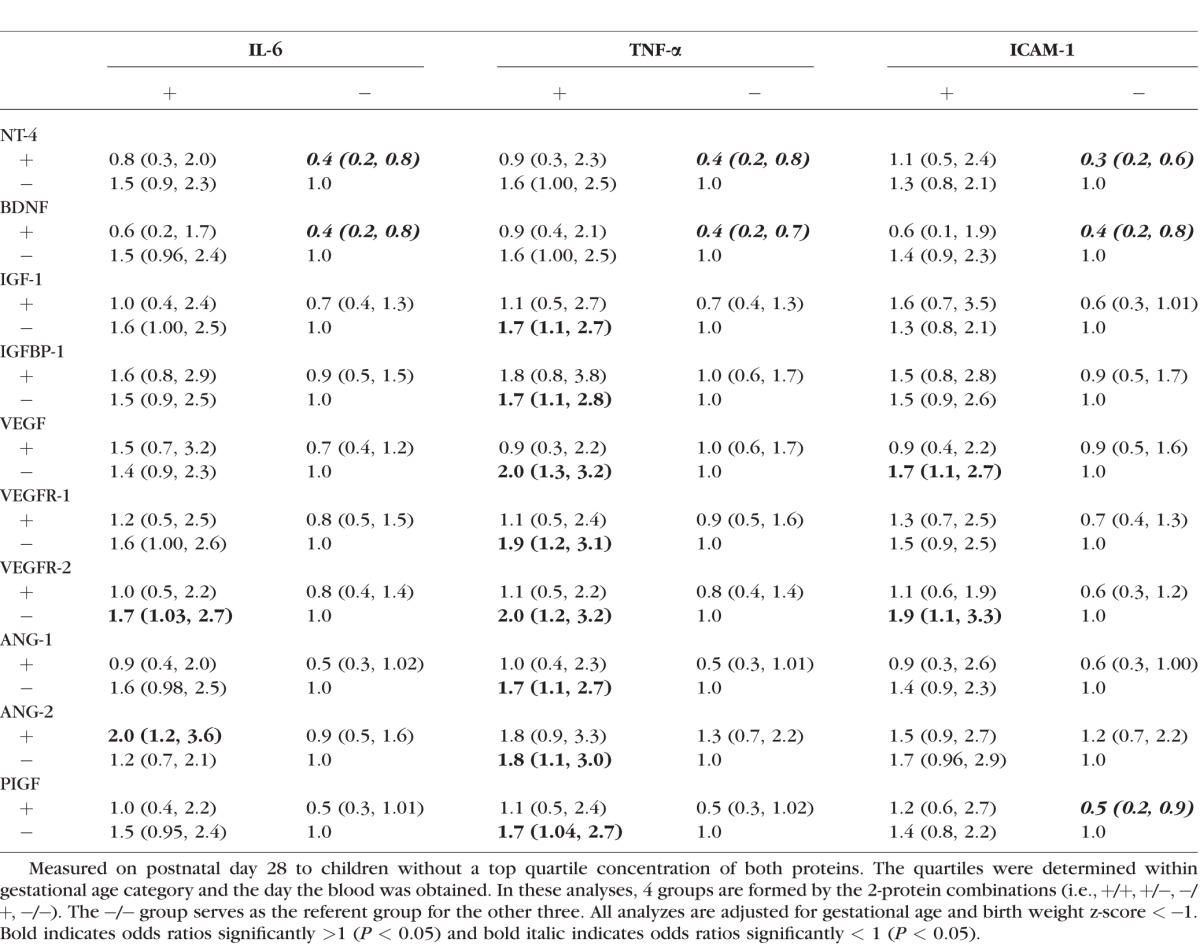
Odds ratios (95% CI) for Prethreshold ROP Comparing Children With (+) and Without (−) a Top Quartile Blood Concentration of Each of Two Proteins

On day 28, the ROP risk was reduced when the neurotropic factors NT-4 and/or BDNF were in the top quartile, but this risk reduction was observed only when the three inflammation-related proteins, IL-6, TNF-α, and ICAM-1, were in the lower three quartiles. This pattern was also observed for PIGF, but only for the inflammation-related protein ICAM-1. In contrast, when the concentration of TNF-α was in the top quartile, the risk of ROP was significantly elevated only when the concentrations of IGF-1, IGFBP-1, VEGF, VEGFR-1, VEGFR-2, ANG-1, ANG-2, or PIGF were not in the top quartile. A heightened ROP risk was also observed when IL-6 was in the top quartile simultaneously with a top quartile of ANG-2 or VEGFR-2 in the lower three quartiles. Top concentrations of ICAM-1 were associated with increased risk of ROP only when VEGFR-2 concentrations were in the lower 3 quartiles.

### ROP Risk Related to the Co-Occurrence of Proteins on Both Days 21 and 28 (Table 6)

Because persisting or recurrent inflammation appears to be more damaging than just isolated incidences of inflammation,^36,37^ we also studied the relationships between top quartile concentrations on two separate days a week apart. Infants who had top quartile concentrations of NT-4, BDNF, and ANG-1 on both days of the late epoch were at reduced risk of ROP when the selected inflammation-related protein (IL-6, TNF-α, or ICAM-1) was not in the top quartile on both days. Children who had a top quartile concentration of IL-6 on both days were at increased risk of ROP when the concentration of the neurotrophic protein (BDNF, IGF-1, IGFBP-1, VEGF, VEGFR-1 and 2, ANG-1, or PIGF) was not in the top quartile on both days. Similarly, top concentrations of ICAM-1 were associated with increased risk of ROP only when VEGFR-2 concentrations were in the lower three quartiles. A heightened ROP risk was also observed when IL-6 was in the top quartile simultaneously with a top quartile of ANG-2.

**Table 6:**
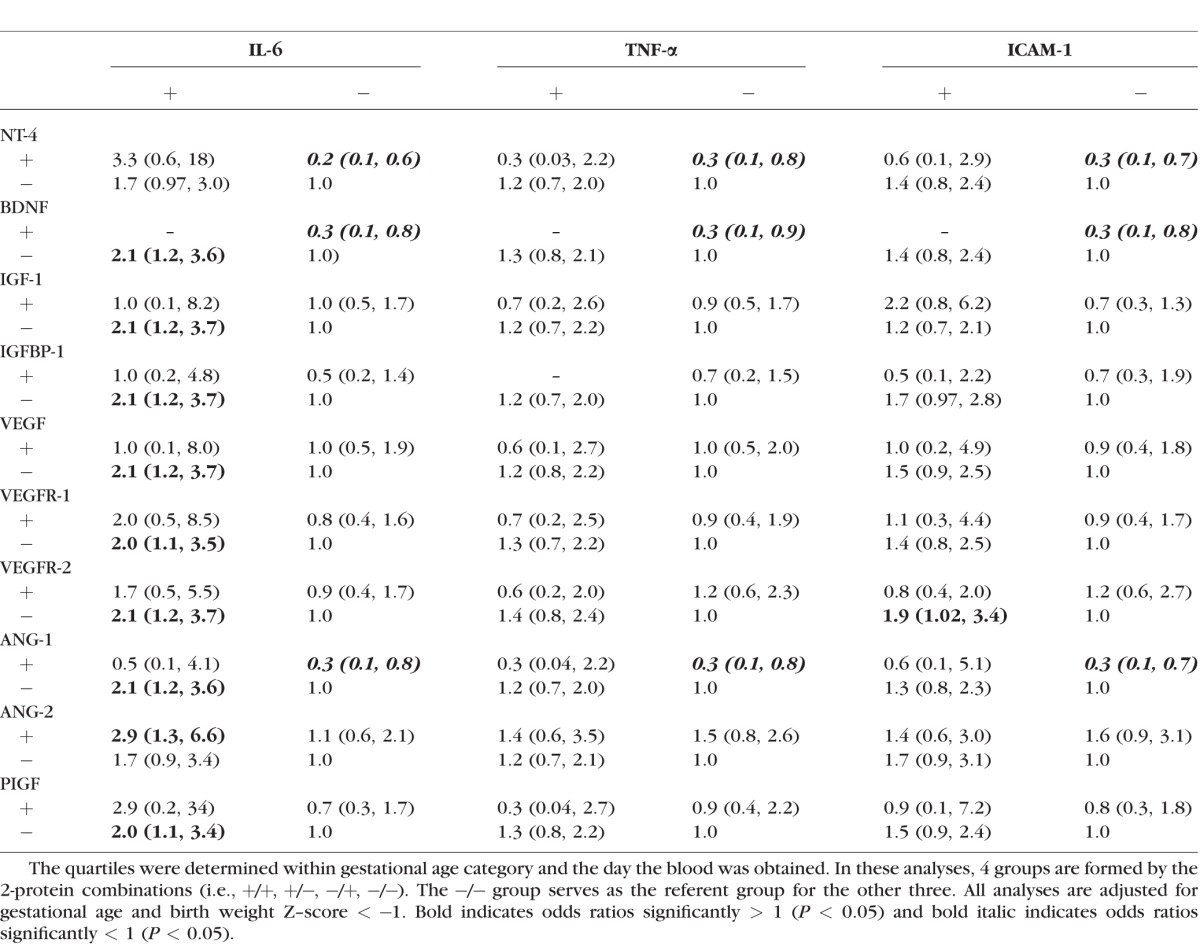
Odds Ratios (95% CI) for Prethreshold ROP Comparing Children With (+) and Without (−) a Top Quartile Blood Concentration of Each of Two Proteins Measured on Both Postnatal Days 21 and 28 to Children Without a Top Quartile Concentration of Both Proteins on Both Days

## Discussion

Three of our findings are worthy of discussion. First, we found an increased ROP risk when some of the inflammation-related proteins were elevated. Second, infants who had top quartile concentrations of proteins with angiogenic and/or neurotrophic properties were at reduced risk of ROP, most prominently in the 3rd and 4th postnatal weeks (the late epoch). Third, there was a modulatory interaction between levels of proinflammatory proteins (e.g., IL-6, TNF-α, and ICAM-1) and a number of neuroprotective proteins. For some proteins (e.g., VEGFR-2, IGF-1) the protective effects were only apparent when we stratified the sample by the co-occurrence of a top concentration of the proteins associated with increased ROP risk (e.g., IL-6, TNF-α, and ICAM-1).

The pathogenesis of ROP is classically described as a 2-phase disruption of the retinal vascular development, where the first phase is characterized by cessation of retinal vessel formation and maturation, while new and aberrant vessel formation and growth occurs in the second phase. Prenatal phenomena have the potential to sensitize the developing retina, and subsequent postnatal stimuli/exposures together might constitute a “prephase” of ROP.^12^

We found that elevated concentrations of inflammatory mediators during the latter part of the first postnatal month (days 14–28) were most predictive of later ROP. As the second phase of ROP begins close to weeks 30 to 32 and the gestational age of the infants in our cohort varies, most of our protein concentration measurements were from specimens obtained before neovascularization begins or very early during neovascularization. Our findings thereby support the concept of a prephase that precedes phase 1.

### Inflammation Is a Risk Factor for Prethreshold ROP

We identified TNF-α, TNF-R1, TNF-R2, IL-6, IL-8, SAA, CRP, ICAM-1, and MPO as proteins with potential to increase ROP risk. SAA and CRP are well-established liver-derived markers of systemic inflammation.^38^ TNF-α, and IL-6 are cytokines acting as primary initiators of inflammation following infection or tissue damage, activating macrophages and T-cells, as well as inducing angiogenesis and apoptosis.^39^ In the retina, TNF-α is involved in proliferative retinopathies as well as inflammatory diseases,^40^ and high plasma concentrations of IL-6, IL-8, and TNF-α have been associated with increased risk of ROP.^17,41^ Soluble TNF-receptors has potential to increase the half-life of TNF-α in the blood circulation.^42^ The inflammatory response includes upregulation of effector molecules such as chemokines (e.g., IL-8, RANTES), and adhesion molecules (e.g., ICAM-1).^43^ Because low vitreous and blood concentrations of RANTES have been found in children who developed severe ROP,^17,44,45^ RANTES might play a protective role. Our finding that high RANTES concentrations were associated with lower risk, although only in the early epoch, supports this inference.

### Neurotrophic Proteins Appear to Protect Against ROP

We found a variety of biomarkers associated with reduced ROP risk, including IGF-1; angiopoietin (ANG-1); and neurotrophins (NT-4 and BDNF). These molecules are referred to as angioneurins, and are capable of both neural and vascular protection and repair.^22^ Top-quartile concentrations of ANG-1 most consistently predicted reduced ROP risk on all days, except day 1. Both ANG-1 and ANG-2 are vascular growth factors important both in fetal life and after birth, where they remodel the developing vasculature. Both are ligands of the Tie2 receptor, although one is in an agonist (ANG-1) and the other an antagonist (ANG-2). Whereas ANG-1 promotes vascular maturation and stability, ANG-2 initiates vessel instability and neovascularization.^46,47^ Our study further emphasizes ANG-1 as a possible protector throughout the whole prephase and phase 1 of ROP.

Neurotrophins belong to a family of growth factors that promote neuronal as well as oligodendrocyte survival and differentiation both in the central and peripheral nervous systems.^48,49^ Although the focus has been on the ability of neurotrophins to protect and repair the neural system, they can also have a beneficial effect on the vascular system.^22,50^ We found that high concentrations of the neurotrophins NT-4 and BDNF were associated with a reduced risk for prethreshold ROP. This is in keeping with other studies where low serum concentrations of NT-4 and BDNF were associated with an increased ROP risk.^17,51^

We found a top quartile concentration of VEGF on day 7 only to be associated with a reduced ROP risk. In the setting of top quartile concentrations of TNF-α, IL-6, and ICAM-1 on days 21 and 28, top quartile concentrations of VEGF on those days were associated with lower risks of ROP than observed when VEGF concentrations were lower. These findings are probably reflective of what is seen in the retina, with suppression of VEGF expression during the first hypoxic phase of ROP, which coincides with the timing of the samples tested in this study. After the first phase, VEGF expression is upregulated and potentiated by growth factors such as IGF-1, resulting in proliferative ROP. It is at this point that intravitreous anti-VEGF agents can be given to effectively treat severe ROP.^52–54^ VEGF production can be stimulated by hypoxia, but systemic diseases, oxygen treatment and respiratory distress might also trigger VEGF production and release.^55^ From this we infer that maintenance of higher VEGF levels during the ROP prephase or phase 1 would be reflective of less vasocessation, and thus might reduce ROP risk.

### Looking at Inflammation and Protection Simultaneously

We examined the risk of ROP associated with concurrent high concentrations of inflammation-associated proteins and high concentrations of proteins with neurotrophic and/or angiogenic properties (angioneurins). This reflects our perception that one can best identify the contribution of an angioneurin in the presence of high concentrations of an inflammation-related protein, and that the contribution of high concentrations of an inflammation-related protein is most easily identified when the concentrations of angioneurins are low.

The most common pattern we found was characterized by an increased ROP risk when the concentration of an inflammation-related protein was elevated, and the concentration of an angioneurin was not. One way to interpret this is that high concentrations of the inflammation-associated protein do not elevate ROP risk if the angioneurins are simultaneously high. Inversely top quartile late epoch concentrations of NT-4 and BDNF appeared protective only in the absence of elevated inflammatory mediators (e.g., IL-6, TNF-α and ICAM-1). Systemic inflammation has the ability to decrease gene expression of BDNF in mice.^56^ In the ELGAN study, infants who developed systemic inflammation were more likely to have higher neurotrophin concentrations than their peers with no systemic inflammation.^57^ Also in this sample, elevated concentrations of VEGF, VEGFR-1, VEGFR-2, PlGF, ANG-1, and ANG-2 were associated with same-day elevated concentrations of inflammation-related proteins, each other, as well as with proteins that have neurotrophic properties.^58^ One explanation for the co-occurrence of elevated concentrations of inflammation-related, angiogenic, and neurotrophic factors (potential protectors) is that a common antecedent upregulates all of them.^59^ Another is that one response (probably inflammation) upregulates the other (protection) as a “self-defense” mechanism. Without additional data about protein response dynamics it is impossible to distinguish between the two options, but we think that the “self-defense” hypothesis is supported by our finding effects of one in the absence of the other (i.e., a risk increase with inflammation in the absence of elevated concentrations of protectors, and a risk decrease with elevated concentrations of protectors in the absence of inflammation; Tables 5 and 6). When analyzed individually, IGF-1, IGFPB-1, and VEGFR-2 showed no evidence of protection (Table 4). However, when high concentrations of these proteins are evaluated in light of the concentrations of IL-6, TNF-α, and ICAM-1, risk modulation was identified. We encourage others to use this strategy. Top concentrations of the three proteins were not risk-defining in the absence of inflammation, but the damage caused by inflammatory proteins appeared to increase ROP risk only when these proteins failed to be elevated. This suggests a primary role in repair, but also the possibility that inflammatory mediators (e.g., IL-6 and TNF-α), downregulate these proteins as one of the mechanisms of inflammation-driven damage to the retina.

IGF-1 is important for fetal growth, including healthy retinal angiogenesis. IGF-1 is also probably necessary for normal VEGF function.^60^ Low systemic serum IGF-1 concentrations are associated with increased risk of ROP, and have been used to identify infants at risk of developing ROP.^60–63^ Hence, exogenous IGF-1 supplements have been suggested as a potential prophylaxis for ROP.^24^ Interestingly, lower plasma concentrations of IGF-1 binding protein (IGFBP-3) were found in infants developing severe ROP.^64^ We did not find elevated concentrations of IGF-1 or IGFBP-1 to reduce the risk of ROP in the absence of inflammation as defined in this study.

We view inflammation as a complex phenomenon that includes many proteins from multiple functional categories affecting multiple systems.^65–67^ The individual inflammation-related proteins measured are probably surrogates for many unmeasured proteins involved in inflammatory processes. The angioneurins included here (including IGF-1) are surrogates for many unmeasured proteins involved in retinal maturation and repair. Consequently, exogenous IGF-1 might not be able to achieve all that is needed to enhance retinal maturation and repair. On the other hand, we did find infants in the lower three IGF-1/IGFBP-1 quartiles to be vulnerable when concentrations of the inflammatory mediators IL-6 and TNF-α where elevated. This is in keeping with the finding that inflammation might be a factor that reduces the already limited IGF-1 production in the preterm newborn.^68^

### Strengths and Limitations

Our study has several strengths. First, we obtained multiple blood spots weekly during the first postnatal month. The multiphased development of ROP underscores the importance of assessing biomarker concentrations longitudinally, as different biomarkers probably play different roles in the different phases of the disease. Second, we measured the concentrations of a diverse set of proteins, including cytokines, chemokines, adhesion molecules, a matrix metalloproteinase, and a diverse set of growth factors. Third, we included more than 1000 infants, making it unlikely that we have missed important associations due to lack of statistical power, or claimed associations that might have reflected the instability of small numbers. Fourth, the infants were selected based on gestational age, not birth weight, in order to minimize confounding due to factors related to fetal growth restriction.^69^ Fifth, we collected all of our data prospectively, thereby minimizing bias. Finally, our protein data are of high quality,^70,71^ and have high content validity.^30,72–74^

Our study also has limitations. As the blood–retinal barrier surrounds the eyes, the concentrations of cytokines and growth factors in blood do not necessarily reflect the levels found in the retina and vitreous of the eye.^75^ On the other hand, inflammation appears capable of reducing the effectiveness of these barriers,^76^ making it difficult to estimate to what extent circulating proteins influence the retina directly. Our blood specimens were collected at time points in the early postnatal period, corresponding to the prephase and 1st phase of ROP. Subsequently, any biomarker levels or differences that may occur later, at the time of phase 2 and maximal ROP development, are still unknown.

The blood we measured was not whole blood, plasma, or serum. Rather, we eluted blood that had been dropped or blotted on filter paper. Because some spots are thicker than others, we normalized our measurements to milligram of total protein in the eluent. Since this is not equivalent to measurements made in freshly obtained blood, our measurements are not directly comparable to measurements obtained from clinical laboratories. Therefore, we do not provide picograms of each protein per milligram of total protein. Moreover, we compared the concentrations of each protein among children who are comparable by virtue of gestational age group, the day the specimen was collected, and the year measurements were made. Because the concentrations varied with each of these, offering a number for each concentration would be highly misleading.

In conclusion, we found that blood concentrations of proteins related to inflammation and growth during the first postnatal month convey information about ROP risk. Top quartile concentrations of inflammatory mediators were identified as risk factors for development of prethreshold ROP, while the top quartile concentrations of proteins with known angiogenic and neurotrophic properties were associated with a reduced ROP risk. This study adds to our knowledge of the complex etiology of ROP development, and is, as far as we know, the first study with such a wide variety of systemic biomarkers in a large cohort of extremely low gestational age newborns.

## Supplementary Material

Supplementary Tables

## Appendix

Participating institutions and members in the ELGAN Study Neonatology and Ophthalmology Committees:

Baystate Medical Center, Springfield, Massachusetts, United States: Bhavesh Shah and William Seefeld.

Beth Israel Deaconess Medical Center, Boston, Massachusetts, United States: Camilia R. Martin and Robert Petersen.

Brigham & Women's Hospital, Boston, Massachusetts, United States: Linda J. Van Marter.

DeVos Children's Hospital, Grand Rapids, Michigan, United States: Mariel Portenga and Patrick Droste.

Forsyth Hospital, Baptist Medical Center, Winston-Salem, North Carolina, United States: T. Michael O'Shea and Grey Weaver.

Massachusetts General Hospital, Boston, Massachusetts, United States: Robert Insoft and Anthony Fraioli.

New England Medical Center, Boston, Massachusetts, United States: Cynthia Cole, John Fiascone, and Jay Duker.

North Carolina Children's Hospital, Chapel Hill, North Carolina, United States: Carl Bose and David Wallace.

Sparrow Hospital, Lansing, Michigan, United States: Padmani Karna and Linda Angell.

UMass Memorial Health Center, Worcester, Massachusetts, United States: Francis Bednarek and Robert Gise.

University Health Systems of Eastern Carolina, Greenville, North Carolina, United States: Stephen Engelke and Elaine Price Schwartz.

University of Chicago Hospital, Chicago, Illinois, United States: Michael D. Schreiber and Ahmed Abdelsalam.

William Beaumont Hospital, Royal Oak, Michigan, United States: Daniel Batton, Anthony Capone, and Michael Trese.

Yale-New Haven Hospital, New Haven, Connecticut, United States: Richard Ehrenkranz and Kathleen Stoessel.
