# Supplementary material for: Systemic Inflammation-Associated Proteins and Retinopathy of Prematurity in Infants Born Before the 28th Week of Gestation
Source: Invest Ophthalmol Vis Sci. 2017 Dec;58(14):6419–28. doi: 10.1167/iovs.17-21931 (PMC5736326; doi:10.1167/iovs.17-21931)
Supplement: Supplementary Tables [file iovs-58-13-53_s01-s03.pdf]

## Supplementary tables:

**Table S1.** Cell percent of **prethreshold retinopathy of prematurity** (N=168) for children who had a concentration of the protein on the left in the **highest quartile** on the postnatal day listed at the top of each column to children who had a concentration that day in the lower three quartiles (i.e., below the 75th centile) (within gestational age category and day blood was obtained), and adjusted for gestational age and birth weight Z-score < -1). The last column of each epoch requires that the elevated concentration be present on 2 of the days in that epoch.

| | | **Early epoch** | | | | **Late epoch** | | |
|---|---|---|---|---|---|---|---|---|
| | | Day 1 N=1107 | Day 7 N=1129 | Day 14 N=1024 | ≥ 2 days N=1141 | Day 21 N=935 | Day28 N=877 | Day 21 & 28 N=766 |
| CRP | + | 12 | 17 | 16 | 15 | 17 | 18 | 24 |
| | − | 15 | 13 | 14 | 14 | 14 | 14 | 14 |
| SAA | + | 10 | 13 | 16 | 13 | 17 | 19 | 28 |
| | − | 16 | 14 | 14 | 14 | 14 | 13 | 14 |
| MPO | + | 13 | 16 | 19 | 17 | 17 | 21 | 21 |
| | − | 14 | 13 | 13 | 13 | 14 | 13 | 14 |
| IL-1β | + | 14 | 13 | 17 | 13 | 16 | 16 | 13 |
| | − | 14 | 14 | 13 | 14 | 14 | 15 | 16 |
| IL-6 | + | 15 | 16 | 17 | 17 | 18 | 20 | 24 |
| | − | 14 | 13 | 14 | 13 | 14 | 13 | 14 |
| IL-6R | + | 13 | 14 | 17 | 16 | 17 | 18 | 20 |
| | − | 14 | 14 | 13 | 13 | 14 | 14 | 14 |
| TNF-α | + | 15 | 14 | 18 | 16 | 19 | 20 | 18 |
| | − | 14 | 14 | 13 | 14 | 13 | 13 | 15 |
| TNF-R1 | + | 15 | 15 | 18 | 18 | 17 | 22 | 23 |
| | − | 14 | 13 | 13 | 13 | 14 | 12 | 14 |
| TNF-R2 | + | 14 | 14 | 17 | 16 | 18 | 20 | 23 |
| | − | 14 | 14 | 13 | 14 | 13 | 13 | 14 |
| IL-8 | + | 13 | 17 | 23 | 21 | 18 | 22 | 23 |
| | − | 15 | 13 | 12 | 13 | 13 | 12 | 14 |
| RANTES | + | 11 | 11 | 10 | 9 | 12 | 12 | 10 |
| | − | 15 | 15 | 16 | 15 | 16 | 16 | 16 |
| ICAM-1 | + | 12 | 16 | 20 | 18 | 18 | 20 | 19 |
| | − | 15 | 13 | 12 | 13 | 13 | 13 | 14 |
| VCAM-1 | + | 13 | 14 | 12 | 13 | 13 | 11 | 15 |
| | − | 15 | 14 | 15 | 14 | 15 | 16 | 15 |
| MMP-9 | + | 13 | 15 | 19 | 16 | 18 | 17 | 22 |
| | − | 14 | 14 | 13 | 14 | 14 | 14 | 14 |
| TSH | + | 15 | 14 | 10 | 11 | 16 | 14 | 19 |
| | − | 14 | 14 | 16 | 15 | 14 | 15 | 15 |
| EPO | + | 14 | 15 | 20 | 18 | 14 | 15 | 17 |
| | − | 14 | 14 | 12 | 13 | 15 | 15 | 15 |

**Table S1, continued.** Cell percent of **prethreshold retinopathy of prematurity** (N=168) for children who had a concentration of the protein on the left in the **highest quartile** on the postnatal day listed at the top of each column to children who had a concentration that day in the lower three quartiles (i.e., below the 75th centile) (within gestational age category and day blood was obtained), and adjusted for gestational age and birth weight Z-score < -1). The last column of each epoch requires that the elevated concentration be present on 2 of the days in that epoch.

| | | **Early epoch** | | | | **Late epoch** | | |
|---|---|---|---|---|---|---|---|---|
| | | Day 1 N=1107 | Day 7 N=1129 | Day 14 N=1024 | ≥ 2 days N=1141 | Day 21 N=935 | Day28 N=877 | Day 21 & 28 N=766 |
| NT-4 | + | 12 | 11 | 14 | 12 | 9 | 9 | 6 |
| | − | 15 | 15 | 14 | 14 | 17 | 17 | 16 |
| BDNF | + | 14 | 13 | 9 | 12 | 9 | 8 | 5 |
| | − | 14 | 14 | 16 | 15 | 17 | 17 | 17 |
| bFGF | + | 15 | 14 | 17 | 15 | 19 | 17 | 20 |
| | − | 14 | 14 | 14 | 14 | 13 | 14 | 15 |
| IGF-1 | + | 14 | 14 | 15 | 13 | 17 | 12 | 14 |
| | − | 14 | 14 | 14 | 14 | 14 | 16 | 15 |
| IGFBP-1 | + | 14 | 17 | 14 | 16 | 14 | 15 | 10 |
| | − | 14 | 13 | 15 | 14 | 15 | 15 | 16 |
| VEGF | + | 13 | 10 | 15 | 11 | 15 | 12 | 13 |
| | − | 15 | 15 | 14 | 15 | 15 | 16 | 15 |
| VEGF-R1 | + | 18 | 15 | 14 | 17 | 16 | 13 | 13 |
| | − | 13 | 13 | 14 | 13 | 14 | 16 | 16 |
| VEGF-R2 | + | 10 | 14 | 16 | 12 | 16 | 13 | 14 |
| | − | 15 | 14 | 14 | 15 | 14 | 16 | 15 |
| PIGF | + | 16 | 12 | 15 | 13 | 12 | 10 | 11 |
| | − | 13 | 15 | 14 | 14 | 16 | 16 | 16 |
| Ang-1 | + | 12 | 10 | 10 | 10 | 10 | 10 | 5 |
| | − | 15 | 15 | 16 | 15 | 16 | 17 | 17 |
| Ang-2 | + | 12 | 14 | 16 | 14 | 17 | 17 | 19 |
| | − | 15 | 14 | 14 | 14 | 14 | 14 | 14 |

**Table S2.** Cell percent of prethreshold retinopathy of prematurity for children with (+) and without (−) a top quartile blood concentration of each of two proteins measured in specimens obtained on **postnatal day 28** to children without a top quartile concentration of both proteins. The quartiles were determined within gestational age category and day blood was obtained.

| | | **IL-6** | | **TNF-α** | | **ICAM-1** | |
|---|---|---|---|---|---|---|---|
| | | + | − | + | − | + | − |
| **NT-4** | + | 14 | 8 | 13 | 7 | 18 | 6 |
| | − | 21 | 15 | 21 | 16 | 20 | 16 |
| **BDNF** | + | 11 | 7 | 13 | 7 | 9 | 8 |
| | − | 22 | 16 | 23 | 15 | 22 | 16 |
| **IGF-1** | + | 14 | 10 | 16 | 11 | 22 | 9 |
| | − | 20 | 14 | 21 | 14 | 19 | 15 |
| **IGFBP-1** | + | 20 | 13 | 19 | 14 | 19 | 13 |
| | − | 19 | 13 | 20 | 13 | 20 | 13 |
| **VEGF** | + | 18 | 10 | 10 | 13 | 12 | 13 |
| | − | 20 | 14 | 23 | 13 | 22 | 13 |
| **VEGFR1** | + | 17 | 11 | 14 | 12 | 17 | 10 |
| | − | 20 | 14 | 23 | 13 | 20 | 14 |
| **VEGFR2** | + | 14 | 12 | 13 | 12 | 16 | 9 |
| | − | 22 | 14 | 24 | 13 | 25 | 14 |
| **Ang-1** | + | 13 | 8 | 13 | 8 | 14 | 8 |
| | − | 21 | 15 | 22 | 15 | 20 | 15 |
| **Ang-2** | + | 26 | 12 | 20 | 15 | 19 | 15 |
| | − | 16 | 13 | 20 | 13 | 20 | 12 |
| **PIGF** | + | 13 | 8 | 14 | 9 | 17 | 8 |
| | − | 22 | 14 | 22 | 15 | 21 | 15 |

**Table S3.** Cell percent of prethreshold retinopathy of prematurity for children with (+) and without (−) a top quartile blood concentration of each of two proteins measured in specimens obtained on **both postnatal days 21 and 28** to children without a top quartile concentration of both proteins on both days. The quartiles were determined within gestational age category and day blood was obtained.

| | | IL-6 | | TNF-α | | ICAM-1 | |
|---|---|---|---|---|---|---|---|
| | | + | − | + | − | + | − |
| **NT-4** | + | 29 | 4 | 5 | 6 | 11 | 5 |
| | − | 24 | 15 | 19 | 16 | 20 | 16 |
| **BDNF** | + | 0 | 5 | 0 | 5 | 0 | 5 |
| | − | 27 | 13 | 21 | 16 | 21 | 16 |
| **IGF-1** | + | 11 | 14 | 12 | 14 | 30 | 10 |
| | − | 26 | 14 | 19 | 15 | 18 | 15 |
| **IGFBP-1** | + | 13 | 9 | 0 | 11 | 8 | 10 |
| | − | 26 | 14 | 19 | 15 | 22 | 15 |
| **VEGF** | + | 9 | 14 | 9 | 14 | 13 | 13 |
| | − | 26 | 14 | 19 | 15 | 20 | 14 |
| **VEGFR1** | + | 33 | 10 | 13 | 13 | 16 | 12 |
| | − | 24 | 14 | 19 | 15 | 19 | 14 |
| **VEGFR2** | + | 21 | 13 | 8 | 17 | 12 | 16 |
| | − | 25 | 14 | 21 | 14 | 23 | 14 |
| **Ang-1** | + | 9 | 4 | 5 | 5 | 10 | 4 |
| | − | 26 | 15 | 20 | 17 | 20 | 17 |
| **Ang-2** | + | 31 | 15 | 20 | 19 | 17 | 21 |
| | − | 21 | 13 | 17 | 14 | 20 | 13 |
| **PIGF** | + | 33 | 10 | 6 | 13 | 9 | 11 |
| | − | 24 | 14 | 19 | 15 | 20 | 15 |
